# Structure and Function of the First Full-Length Murein Peptide Ligase (Mpl) Cell Wall Recycling Protein

**DOI:** 10.1371/journal.pone.0017624

**Published:** 2011-03-18

**Authors:** Debanu Das, Mireille Hervé, Julie Feuerhelm, Carol L. Farr, Hsiu-Ju Chiu, Marc-André Elsliger, Mark W. Knuth, Heath E. Klock, Mitchell D. Miller, Adam Godzik, Scott A. Lesley, Ashley M. Deacon, Dominique Mengin-Lecreulx, Ian A. Wilson

**Affiliations:** 1 Joint Center for Structural Genomics (http://www.jcsg.org); 2 Stanford Synchrotron Radiation Lightsource, SLAC National Accelerator Laboratory, Menlo Park, California, United States of America; 3 Université Paris-Sud, Laboratoire des Enveloppes Bactériennes et Antibiotiques, Orsay, France; 4 Centre National de la Recherche Scientifique, Institut de Biochimie et Biophysique Moléculaire et Cellulaire, Orsay, France; 5 Protein Sciences Department, Genomics Institute of the Novartis Research Foundation, San Diego, California, United States of America; 6 Department of Molecular Biology, The Scripps Research Institute, La Jolla, California, United States of America; 7 Center for Research in Biological Systems, University of California San Diego, La Jolla, California, United States of America; 8 Program on Bioinformatics and Systems Biology, Sanford-Burnham Medical Research Institute, La Jolla, California, United States of America; National Institute for Medical Research, Medical Research Council, United Kingdom

## Abstract

Bacterial cell walls contain peptidoglycan, an essential polymer made by enzymes in the Mur pathway. These proteins are specific to bacteria, which make them targets for drug discovery. MurC, MurD, MurE and MurF catalyze the synthesis of the peptidoglycan precursor UDP-*N*-acetylmuramoyl-L-alanyl-γ-D-glutamyl-*meso*-diaminopimelyl-D-alanyl-D-alanine by the sequential addition of amino acids onto UDP-*N*-acetylmuramic acid (UDP-MurNAc). MurC-F enzymes have been extensively studied by biochemistry and X-ray crystallography. In Gram-negative bacteria, ∼30–60% of the bacterial cell wall is recycled during each generation. Part of this recycling process involves the murein peptide ligase (Mpl), which attaches the breakdown product, the tripeptide L-alanyl-γ-D-glutamyl-*meso*-diaminopimelate, to UDP-MurNAc. We present the crystal structure at 1.65 Å resolution of a full-length Mpl from the permafrost bacterium *Psychrobacter arcticus* 273-4 (*Pa*Mpl). Although the Mpl structure has similarities to Mur enzymes, it has unique sequence and structure features that are likely related to its role in cell wall recycling, a function that differentiates it from the MurC-F enzymes. We have analyzed the sequence-structure relationships that are unique to Mpl proteins and compared them to MurC-F ligases. We have also characterized the biochemical properties of this enzyme (optimal temperature, pH and magnesium binding profiles and kinetic parameters). Although the structure does not contain any bound substrates, we have identified ∼30 residues that are likely to be important for recognition of the tripeptide and UDP-MurNAc substrates, as well as features that are unique to *Psychrobacter* Mpl proteins. These results provide the basis for future mutational studies for more extensive function characterization of the Mpl sequence-structure relationships.

## Introduction

Bacterial cell walls are characterized by the presence of peptidoglycan (murein), a macromolecule built from sugar and peptide building blocks, with the sugars in an alternating arrangement of *N*-acetylglucosamine (GlcNAc) and *N*-acetylmuramic acid (MurNAc). This peptidoglycan barrier helps to maintain turgidity of the cell and balance the internal osmotic pressure [1]. Disruption of proper bacterial cell wall formation is an effective strategy for combating bacterial infections using common antibiotics, such as penicillins [2].

**Figure 1:**
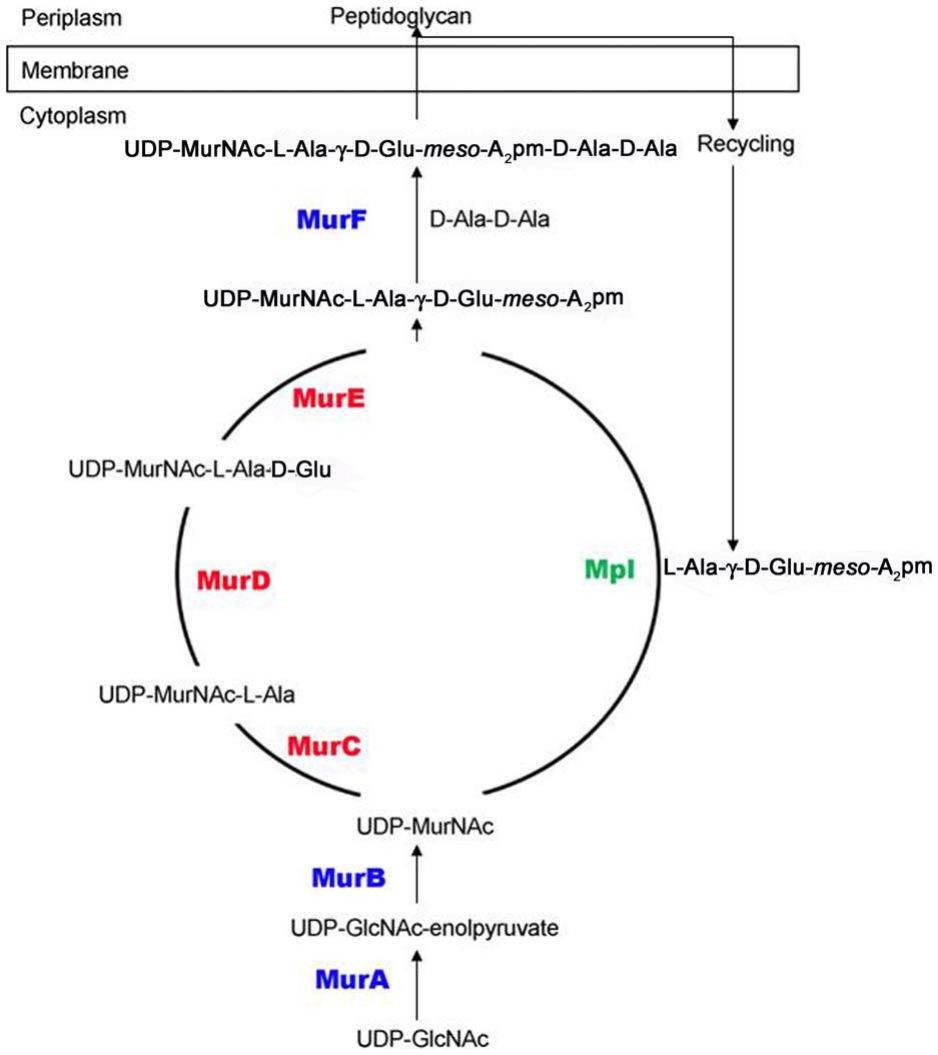
Bacterial cell wall peptidoglycan synthesis. Schematic pathway of cell wall peptidoglycan metabolism adapted from Mengin-Lecreulx *et al.* [6]. Enzymes MurC, MurD and MurE are involved in *de novo* synthesis of the bacterial cell wall peptidoglycan. In Gram-negative bacteria, it is estimated that ∼30–60% of the peptidoglycan is recycled, and involves the enzyme Mpl.

*De novo* peptidoglycan synthesis is carried out by a series of enzymes that have been extensively studied biochemically and structurally [3]–[5]. Briefly, MurA and MurB are involved in the first steps of peptidoglycan assembly with the formation of UDP-GlcNAc-enolpyruvate and UDP-MurNAc, respectively. The Mur ligases, MurC, MurD, MurE and MurF, are then involved in sequential addition of L- and D- amino acids [L-Ala, D-Glu, *meso*-diaminopimelate (*meso*-A_2_pm) and D-Ala-D-Ala, respectively] to form UDP-MurNAc-L-Ala-γ-D-Glu-*meso*-A_2_pm-D-Ala-D-Ala (Figure 1)[6]. This polymer is further processed by several membrane and periplasmic enzymes to ultimately create the peptidoglycan. MurC-F enzymes share a common reaction mechanism, similar three-dimensional structures, and several invariant residues that classifies them in the Mur ligase family [7]–[10].

*Recycling* of the bacterial cell wall is believed to account for ∼30–60% of peptidoglycan synthesis in Gram-negative bacteria [11]. During this process, the cell wall is catabolized using transglycosidases, amidases, endopeptidases and carboxypeptidases [12]. The Mpl protein is used to link the breakdown component, the tripeptide L-Ala-γ-D-Glu-*meso*-A_2_pm (and also tetra- and pentapeptides), to UDP-MurNAc (Figure 1). Mpl was first identified by sequence similarity with MurC (∼20–25% identity) [6] and was also, therefore, assigned to the Mur ligase family. Mpl is functionally similar to MurC, as both mechanisms involve ligation of an amino acid or peptide to UDP-MurNAc; in MurC, a single amino acid (L-Ala) is attached and in Mpl, a tri-, tetra- or pentapeptide.

Mpl from *Escherichia coli* (*Ec*Mpl, 48% identity to *Pa*Mpl) has been functionally characterized [13]. The first 3D structural information was obtained from a truncated Mpl protein (2 out of 3 domains: UDP-MurNAc and ATP binding domains) from *Neisseria meningitidis* (*Nm*Mpl, Midwest Center for Structural Genomics, http://www.mcsg.org, PDB accession code 3eag, 54% sequence identity to *Pa*Mpl, unpublished results). In contrast, numerous crystal structures of the full-length MurC-F proteins (∼15–25% identity to *Pa*Mpl) have been determined, including *Escherichia coli* MurD (*Ec*MurD), *E. coli* MurE (*Ec*MurE) and *Mycobacterium tuberculosis* MurE, and *E. coli* MurF (*Ec*MurF) and *Streptococcus pneumoniae* MurF [10], [14], [15]. The numerous crystal structures of MurC from different bacteria include apo forms of MurC from *E. coli* (*Ec*MurC, PDB 2f00 [16], 25% seq id to *Pa*Mpl), *Haemophilus influenzae* MurC (*Hi*MurC, PDB 1gqq, 25% seq id to *Pa*Mpl), *Thermotoga maritima* MurC (*Tm*MurC, PDB 1j6u [17], 21% seq id to *Pa*Mpl), and ligand-bound MurC structures of *Hi*MurC (PDB 1p3d and 1p31 [18] and 1gqy).

In this study, we have determined the crystal structure of a full-length apo-Mpl protein from the permafrost bacterium *Psychrobacter arcticus* 273-4, *Pa*Mpl (UniProt accession code Q4FVQ2_PSYA2, locus name Psyc_0032, 505 amino acids) at 1.65 Å resolution and have also biochemically characterized this enzyme. We have analyzed the sequence features that differentiate Mpl from MurC-F as well as the common regions shared by different Mpl enzymes. Several features appear to be unique to Mpl from *Psychrobacter* species that may be of importance to permafrost bacteria.

## Materials and Methods

### Protein production and crystallization

Clones were generated using the Polymerase Incomplete Primer Extension (PIPE) cloning method [19]. The gene encoding Mpl (GenBank: YP_263340, gi|71064613; UniProt: Q4FVQ2) was amplified by polymerase chain reaction (PCR) from *P. arcticus* 273-4 genomic DNA using *PfuTurbo* DNA polymerase (Stratagene) and I-PIPE (Insert) primers that included sequences for the predicted 5' and 3' ends. The expression vector, pSpeedET, which encodes an amino-terminal tobacco etch virus (TEV) protease-cleavable expression and purification tag (MGSDKIHHHHHHENLYFQ/G), was PCR amplified with V-PIPE (Vector) primers. V-PIPE and I-PIPE PCR products were mixed to anneal the amplified DNA fragments together. *E. coli* GeneHogs (Invitrogen) competent cells were transformed with the I-PIPE/V-PIPE mixture and dispensed on selective LB-agar plates. The cloning junctions were confirmed by DNA sequencing.

Expression was performed in selenomethionine-containing medium at 310 K. Selenomethionine was incorporated *via* inhibition of methionine biosynthesis [20], which does not require a methionine-auxotrophic strain. At the end of fermentation, lysozyme was added to the culture to a final concentration of 250 µg/ml, and the cells were harvested and frozen. After one freeze/thaw cycle, the cells were sonicated in lysis buffer [50 mM HEPES, pH 8.0, 50 mM NaCl, 10 mM imidazole, 1 mM Tris(2-carboxyethyl)phosphine-HCl (TCEP)] and the lysate was clarified by centrifugation at 32,500× *g* for 30 min. The soluble fraction was passed over nickel-chelating resin (GE Healthcare) pre-equilibrated with lysis buffer, the resin washed with wash buffer [50 mM HEPES, pH 8.0, 300 mM NaCl, 40 mM imidazole, 10% (v/v) glycerol, 1 mM TCEP], and the protein eluted with elution buffer [20 mM HEPES, pH 8.0, 300 mM imidazole, 10% (v/v) glycerol, 1 mM TCEP]. The eluate was buffer exchanged with TEV buffer [20 mM HEPES, pH 8.0, 200 mM NaCl, 40 mM imidazole, 1 mM TCEP] using a PD-10 column (GE Healthcare), and incubated with 1 mg of TEV protease per 15 mg of eluted protein. The protease-treated eluate was run over nickel-chelating resin (GE Healthcare) pre-equilibrated with HEPES crystallization buffer [20 mM HEPES, pH 8.0, 200 mM NaCl, 40 mM imidazole, 1 mM TCEP] and the resin was washed with the same buffer. The flow-through and wash fractions were combined and concentrated for crystallization trials to 12.8 mg/ml by centrifugal ultrafiltration (Millipore). *Pa*Mpl was crystallized using the nanodroplet vapor diffusion method [21] with standard JCSG crystallization protocols [22]. Sitting drops composed of 200 nl protein mixed with 200 nl crystallization solution were equilibrated against a 50 µl reservoir at 277 K for 26 days prior to harvest. The crystallization reagent that produced the *Pa*Mpl crystal for structure determination consisted of 0.2 M sodium acetate, 30% PEG-4000, and 0.1 M Tris-HCl, pH 8.5. No cryoprotectant was added to the crystal. Initial screening for diffraction was carried out using the Stanford Automated Mounting system (SAM) [23] at the Stanford Synchrotron Radiation Lightsource (SSRL, SLAC National Accelerator Laboratory, Menlo Park, CA). The diffraction data were indexed in monoclinic space group C2. The oligomeric state of *Pa*Mpl in solution was determined using a 1×30 cm Superdex 200 column (GE Healthcare) coupled with miniDAWN static light scattering (SEC/SLS) and Optilab differential refractive index detectors (Wyatt Technology). The mobile phase consisted of 20 mM Tris-HCl pH 8.0, 150 mM sodium chloride, and 0.02% (w/v) sodium azide. The molecular weight was calculated using ASTRA 5.1.5 software (Wyatt Technology).

### X-ray data collection, structure solution, and refinement

MAD data were collected at the SSRL on beamline 9–2 at wavelengths corresponding to the high-energy remote (λ_1_), inflection point (λ_2_) and peak (λ_3_) of a selenium MAD experiment using the BLU-ICE [24] data collection environment. The data sets were collected at 100 K using a MarMosaic 325 CCD detector (Rayonix, USA). The MAD data were integrated and reduced using MOSFLM [25] and scaled with the program SCALA [26]. The heavy atom sub-structure was determined with SHELXD [27]. Phasing was performed with autoSHARP [28], SOLOMON [29] (implemented in autoSHARP) was used for density modification and ARP/wARP [30] was used for automatic model building to 1.65 Å resolution. Model completion and crystallographic refinement were performed with the λ_1_ dataset using COOT [31] and REFMAC5 [32]. The refinement protocol included experimental phase restraints in the form of Hendrickson–Lattman coefficients from autoSHARP and TLS refinement with one TLS group per domain. Data and refinement statistics are summarized in Table 1 [33].

**Table 1:** Summary of crystal parameters, data collection and refinement statistics for PDB 3hn7.

Space group	C2		
Unit cell parameters	a = 105.45 Å, b = 52.76 Å, c = 89.99 Å, β = 98.47°		
**Data collection**	λ_1_ MAD-Se	λ_2_ MAD-Se	λ_3_ MAD-Se
Wavelength (Å)	0.9116	0.9793	0.9792
Resolution range (Å)	29.0–1.65	29.0–1.65	29.0–1.69
Number of observations	196,918	195,361	182,672
Number of unique reflections	59,026	58,985	55,004
Completeness (%)	100 (100)^a^	100 (100)^a^	100 (100)^a^
Mean I/σ(I)	11.5 (2.2)^a^	9.9 (1.7)^a^	10.5 (1.8)^a^
Rsym on I (%)	0.076 (0.51)^a^	0.093 (0.58)^a^	0.085 (0.54)^a^
Highest resolution shell (Å)	1.69–1.65	1.69–1.65	1.73–1.69
**Model and refinement statistics**			
Resolution range (Å)	29.0–1.65	Data set used in refinement	λ_1_ MAD-Se
No. of reflections (total)	59,024^b^	Cutoff criteria	|F|>0
No. of reflections (test)	2,985	R_cryst_	0.154
Completeness (% total)	100	R_free_	0.187
**Stereochemical parameters**			
Restraints (RMSD observed)			
Bond angle (°)	1.59		
Bond length (Å)	0.018		
Average protein isotropic B-value (Å^2^)	19.6^c^		
ESU based on R_free_ (Å)	0.087		
No. of protein residues/atoms	480/3728		
No. of water molecules	588 (with 10 modeled in alternate positions)		

a Highest resolution shell.

ESU = Estimated overall coordinate error [33].

R_sym_ = Σ|I_i_-<I_i_>|/Σ|I_i_|, where I_i_ is the scaled intensity of the i^th^ measurement and <I_i_> is the mean intensity for that reflection.

R_cryst_ = Σ||F_obs_|-|F_calc_||/Σ|F_obs_|, where F_calc_ and F_obs_ are the calculated and observed structure factor amplitudes, respectively.

R_free_ = as for R_cryst_, but for 5.1% of the total reflections chosen at random and omitted from refinement.

b Typically, the number of unique reflections used in refinement is slightly less that the total number that were integrated and scaled. Reflections are excluded due to negative intensities and rounding errors in the resolution limits and cell parameters.

c This value represents the total B that includes TLS and residual B components.

### Validation and deposition

The quality of the crystal structure was analyzed using the JCSG Quality Control server (http://smb.slac.stanford.edu/jcsg/QC). This server verifies: the stereochemical quality of the model using AutoDepInputTool [34], MolProbity [35], and WHATIF 5.0 [36]; agreement between the atomic model and the data using SFcheck 4.0 [37] and RESOLVE [38], the protein sequence using CLUSTALW [39], atom occupancies using MOLEMAN2 [40], consistency of NCS pairs, and evaluates difference in R_cryst_/R_free_, expected R_free_/R_cryst_ and maximum/minimum B-factors by parsing the refinement log-file and PDB header. Protein quaternary structure analysis was performed using the PISA server [41]. The depiction of the protein sequence on the secondary structure was adapted from an analysis using PDBsum [42], and all other renditions of the protein structure were prepared with PyMOL [43]. Atomic coordinates and experimental structure factors for Mpl from *P. arcticus* 273-4 to 1.65 Å resolution (code 3hn7) have been deposited in the Protein Data Bank (www.wwpdb.org).

### Preparation of nucleotide precursors and peptides

The nucleotide precursor UDP-MurNAc was chemically synthesized [44] and UDP-MurNAc-peptides were prepared as described previously [45]. Radiolabeled UDP-[^14^C]MurNAc was prepared from UDP-[^14^C]GlcNAc (11.5 GBq/mmol, Amersham Biosciences), PEP, NADPH and purified His_6_-tagged MurA and MurB enzymes, as described previously [9]. The dipeptide L-Ala-D-Glu was synthesized chemically according to Sachs and Brand [46] and the tripeptide L-Ala-γ-D-Glu-L-Lys was synthesized by a modified procedure of Schmidt *et al.* [47]. The tetrapeptide L-Ala-γ-D-Glu-L-Lys-D-Ala was generated by treatment of pentapeptide L-Ala-γ-D-Glu-L-Lys-D-Ala-D-Ala (Bachem) with purified *E. coli* PBP5, as described earlier [48]. The latter enzyme was over-expressed as a soluble His_6_-tagged form from the pET-PBP5s plasmid and purified on Ni^2+^-NTA agarose using standard procedures. *meso*-A_2_pm-containing peptides were prepared from the corresponding UDP-MurNAc-peptides as follows: first, mild acid hydrolysis (10 min at 100°C in 0.1 M HCl) of UDP-MurNAc-peptides generated MurNAc-peptides, which were purified by HPLC as mixtures of the two anomers α and β [49]; then, MurNAc-peptides were hydrolyzed to MurNAc and peptides by incubation with purified *N*-acetylmuramoyl-L-alanine amidase (*E. coli* AmiD) [50]. The released peptides were subsequently purified by HPLC on a 3 µm ODS-Hypersil column (0.46×25 cm). Elution was with 0.05% trifluoroacetic acid for the *meso*-A_2_pm-containing tripeptide, or with a gradient of acetonitrile (from 0 to 20% in 30 min) in the same buffer for other peptides, at a flow rate of 0.5 ml/min. Peptides were quantified by amino acid analysis with a Hitachi L8800 analyzer (ScienceTec) after hydrolysis in 6 M HCl for 16 h at 95°C.

### Protein production for biochemical characterization

The pSpeedET:*Pampl* plasmid was used for over-expression of the His_6_-tagged *Pa*Mpl protein in *E. coli* cells. In order to avoid any potential contamination with the *Ec*Mpl protein, the *E. coli* strain MLD2502, which has a deletion of the chromosomal *mpl* gene (BW25113 Δ*mpl*::Cm^R^) [13], was used as the host strain for these experiments. Cells were grown at 37°C in 1 l of 2YT medium (BioRad) supplemented with chloramphenicol and kanamycin (25 and 50 µg/ml, respectively). Growth was monitored at 600 nm and the expression of Mpl was induced with 0.02% (w/v) arabinose when the optical density of the culture reached 0.7. Overproduction of *Pa*Mpl was observed following induction of cultures with arabinose, as judged by SDS-PAGE analysis of cell extracts performed as previously described using 13% polyacrylamide gels [51]. However, amounts of this protein recovered in the supernatant fraction following cell disruption and centrifugation were lower than those recovered previously with the *Ec*Mpl ortholog [13] (data not shown). Cells were harvested 3 h later and washed with cold 20 mM potassium phosphate buffer, pH 7.4, containing 0.5 mM MgCl_2_ and 0.01% 2-mercaptoethanol (buffer A). The cell pellet was resuspended in 10 ml of buffer A containing protease inhibitors (complete EDTA-free, Roche) and disrupted by sonication. The suspension was then centrifuged at 200,000× *g* with a Beckman TL100 centrifuge and the His_6_-tagged Mpl protein present in the soluble fraction (supernatant) was purified at 4°C on Ni^2+^-nitrilotriacetate (Ni^2+^-NTA) agarose (Qiagen). Binding of Mpl protein to the polymer was followed by extensive washing with 45 ml of buffer A containing 300 mM KCl and 10 mM imidazole, and elution was done in 15 ml using a discontinuous gradient of imidazole, from 10 to 300 mM. The His_6_-tagged Mpl was eluted with 100 mM imidazole. After dialysis of the corresponding fraction against 200 volumes of buffer A, the Mpl preparation was applied onto 0.25 ml of cobalt resin (TALON, Clontech) for a further purification-concentration step to eliminate a 30-kDa contaminating protein frequently co-purified with His-tagged proteins, which likely is the SlyD protein [52]. After washing with buffer A containing 10 mM imidazole, the His_6_-tagged Mpl protein was eluted with 0.5 ml of buffer A containing 200 mM imidazole. The final purified fraction was dialyzed against 100 volumes of buffer A and stored at −20°C after addition of glycerol (10% final concentration). The final preparation of *Pa*Mpl was concentrated to 1.2 mg/ml and was at least 95% pure as judged by SDS-PAGE analysis. Mpl migrated as a protein of ∼57 kDa, consistent with the calculated value (57,187 Da, including the His-tag). The yield was relatively good at ∼2 mg/liter of culture, which is, nevertheless, ∼50 fold lower than that routinely obtained with *Ec*Mpl. Protein concentrations were determined by the Bradford method, using bovine serum albumin as a standard [53], or by quantitative amino acid analysis after hydrolysis of a sample in 6.0 M HCl for 24 h at 105°C. To compare the oligomeric form of *Pa*Mpl determined by SEC/SLS (see ***Protein production and crystallization***) to that of *Ec*Mpl, 100 µl of 1.3 mg of *Ec*Mpl was loaded onto a Superdex 200 column (10/300 GL, GE Healthcare). The mobile phase consisted of 20 mM potassium phosphate buffer pH 7.4, 0.15 mM NaCl, 0.01% 2-mercaptoethanol. The velocity was 0.5 ml/min and protein elution was detected at 280 nm.

### Assay for tripeptide ligase activity

The standard assay mixture (40 µl) contained 100 mM Tris-HCl buffer, pH 8.4, 5 mM ATP, 5 mM MgCl_2_, 0.25 mM L-Ala-γ-D-Glu-*meso-*A_2_pm, 0.4 mM UDP-[^14^C]MurNAc (500 Bq), and purified *Pa*Mpl enzyme (70 ng of protein). Mixtures were incubated for 30 min and reactions were stopped by the addition of 10 µl of acetic acid, followed by lyophilization. The residues were dissolved in 150 µl of 50 mM ammonium formate, pH 3.2, and 130 µl-aliquots were injected onto a Nucleosil 100C_18_ 5 µm column (0.46×15 cm, Alltech-France) using the same buffer at 0.6 ml/min as the mobile phase. Detection was performed with a radioactive flow detector (model LB506-C1, Berthold) using the Quicksafe Flow 2 scintillator (Zinsser Analytic) at 0.6 ml/min. Quantitation was carried out with the Radiostar software (Berthold).

For determination of the kinetic constants, the same assay was used with various concentrations of one substrate and fixed concentrations of the others. In all cases, the substrate consumption was <20%, the linearity being ensured within this time interval even at the lowest substrate concentration. The data were fitted to the equation *v* = *V*_max_ *S*/(*K_m_*+*S*) using the MDFitt software developed by M. Desmadril (IBBMC, Orsay, France).

Identical assay conditions were used when other peptides were tested as substrates. The buffer and pH used for the separation of the radiolabeled substrate and product slightly varied depending on substrate used: ammonium formate at pH 3.9 for L-Ala-D-Glu, ammonium formate at pH 4.2 for L-Ala-γ-D-Glu-*meso-*A_2_pm-D-Ala, L-Ala-γ-D-Glu-*meso-*A_2_pm-D-Ala-D-Ala, L-Ala-γ-D-Glu-L-Lys and L-Ala-γ-D-Glu-L-Lys-D-Ala, and 50 mM ammonium acetate at pH 5.0 for L-Ala-γ-D-Glu-L-Lys-D-Ala-D-Ala.

## Results

### Sequence analysis

**Figure 2:**
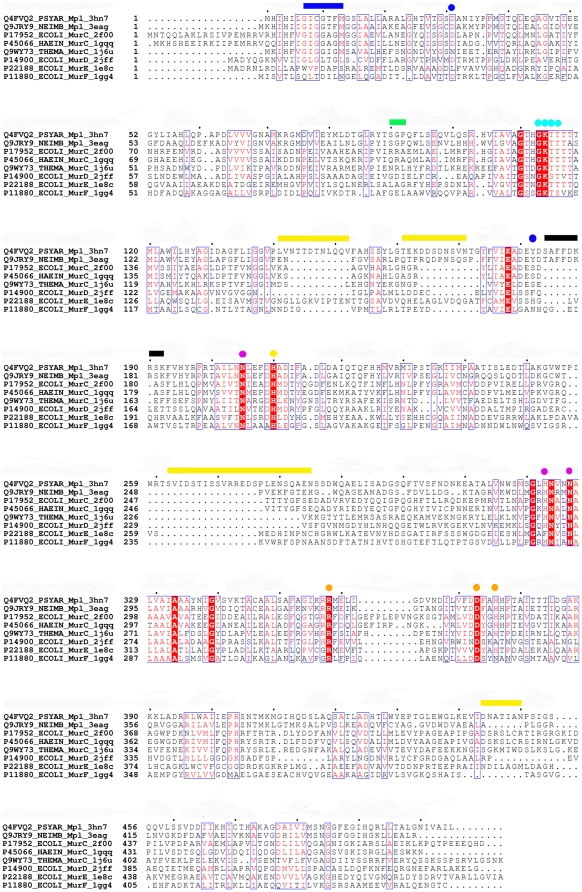
Sequence comparison of Mpl to MurC-F enzymes. Multiple sequence alignment (CLUSTALW [55]) of Mpl with MurC-F, rendered using ESPRIPT [56]. The pairwise sequence identity between Mpl and MurC is ∼21–25%, and is less between Mpl and MurD-F, ∼15–17%. The *Pa*Mpl segment of residues 184–192 (black bar), comprised of the sequence motif (S/T)AFFDKRSK, is not present in Mur ligases and may be functionally important in Mpl. This motif packs against residues 90–92 (green bar), which are unique to and conserved in Mpl proteins (from analysis of 17 annotated Mpl enzymes in the UniProt database, August 2009, data not shown). Residues 7–13 constituting the GI(C/G)GTFM motif (blue bar) and Asp31 (blue circle) are likely to interact with UDP. Tyr182 (blue circle) is unique to Mpl and may be involved in substrate recognition. Residues that are likely to be important in ATP binding are: Asn205, His323 and Asn327 (adenine-binding, magenta circles); Asp373 (ribose-binding, orange circle); and Gly113, Lys114, Thr115, Thr116, Arg358 and His376 (tri-phosphate binding, cyan and orange circles). Metal-binding residues are likely to be Thr115, Glu178 and His210. The *Psychrobacter* Mpl enzymes have several insertions (residues 141–152, 161–171, 262–285 and 444–450, yellow bars), which may be related to cell wall recycling in these bacteria that survive in permafrost conditions.

Mpl and MurC-F enzymes have three domains that are classified in Pfam families [54], PF01225 (Mur ligase catalytic domain), PF08245 (Mur ligase middle domain) and PF02875 (Mur ligase glutamate binding/C-terminal domain). Mpl was reported to exhibit significant sequence similarity with MurC when first isolated and identified [6]. A multiple sequence alignment (calculated using CLUSTALW [55] and depicted using ESPRIPT [56]) between *Pa*Mpl, *Nm*Mpl, *Ec*MurC, *Hi*MurC, *Tm*MurC with MurD-F (*Ec*MurD, *Ec*MurE and *Ec*MurF), shows that significantly more residues are, indeed, conserved between Mpl and MurC (∼21–25% overall pair-wise sequence-based identity) than between Mpl and MurD-F (∼15–17% overall pair-wise sequence identity) (Figure 2). The highest variation is found in the C-terminal, peptide-binding domain. Functionally, Mpl and MurC cannot substitute for each other under normal physiological conditions. However, overexpression of the *E. coli mpl* gene on a multi-copy plasmid was shown to complement an *E. coli* thermosensitive *murC* mutant, which indicated that Mpl could also accept L-alanine as a substrate [13]. The sequence motif (S/T)AFFDKRSK (residues 184–192 in *Pa*Mpl numbering), which is conserved in all 17 annotated Mpl enzymes in the August 2009 UniProt database, is not present in other Mur ligases (Figure 2, black bar). In addition, in these 17 Mpl enzymes, several insertions (residues 141–152, 161–171, 262–285 and 444–450, Figure 2, yellow bars) are found only in *Psychrobacter* Mpl enzymes (n.b. only residues corresponding to 161–171 are also found in the *Nm*Mpl).

### Overall structure

**Figure 3:**
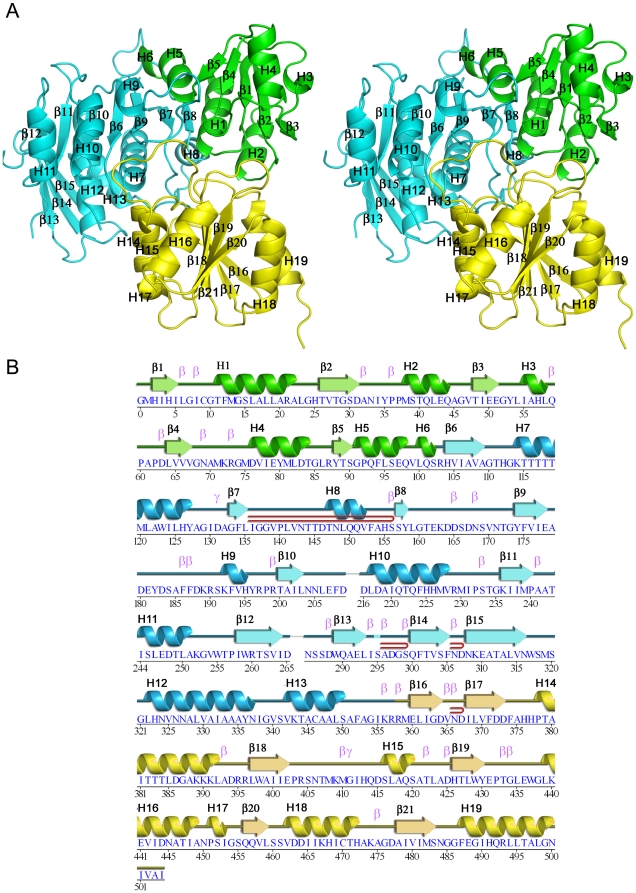
Crystal structure of *Pa*Mpl. (A) Stereo ribbon diagram of *Pa*Mpl highlighting the 3 domains: N-terminal domain (ND), green; Middle Domain (MD), cyan; and C-terminal Domain (CD), yellow. (B) Diagram showing the secondary structure elements of *Pa*Mpl colored by domain and superimposed onto its primary sequence, adapted from PDBsum (http://www.ebi.ac.uk/pdbsum), where α-helices and 3_10_-helices are sequentially labeled (H1, H2, H3 etc), β-strands are labeled (β1, β2, β3, etc), β-hairpins are indicated by red loops, and β- and γ-turns are indicated as β and γ.

The cloning, expression, purification and crystallization of *Pa*Mpl was carried out using standard Joint Center for Structural Genomics (JCSG; http://www.jcsg.org) protocols as detailed in Materials and Methods. The crystal structure of *Pa*Mpl was determined by Multi-wavelength Anomalous Diffraction (MAD) phasing to a resolution of 1.65 Å. Data collection, model and refinement statistics are summarized in Table 1. *Pa*Mpl is present as a monomer in the crystal asymmetric unit (Figure 3). The final model includes Gly0 (a remnant from cleavage of the expression and purification tag); residues 1–209, 216–265, and 285–504 of the protein (the full-length protein contains 505 residues); and 588 water molecules. Residues 210–215, 266–284 and 505 are disordered in the crystal structure and have not been modeled. The Matthews' coefficient [57] is ∼2.2 Å^3^/Da, with an estimated solvent content of ∼45%. The Ramachandran plot produced by Molprobity [35] shows that 98.1% of the residues are in the favored regions with none in disallowed regions.

*Pa*Mpl can be divided into 3 distinct domains: the N-terminal UDP-MurNAc-binding domain (ND, residues 1–102), the middle ATP-binding domain (MD, residues 103–357) and the C-terminal tripeptide-binding domain (CD, residues 358–505). The ND resembles a Rossmann-type fold with a five-stranded, parallel β-sheet (β1–β5) flanked by five α-helices (H1–H5) and 3_10_-helix H6. The MD is a ten-stranded, curved, mainly parallel β-sheet (β6–β15) flanked by helices on each side (α-helices H7-H8 and H10-H13, and 3_10_-helix H9). The CD is a six-stranded, mainly parallel β-sheet (β16–β21) flanked on one side by α-helices H14-H16 and 3_10_-helix H17, and on the other by H18–H19. These 3 domains are linked contiguously to form a triangular-shaped molecule with dimensions of ∼56×60×47 Å^3^.

### Structure comparisons

**Figure 4:**
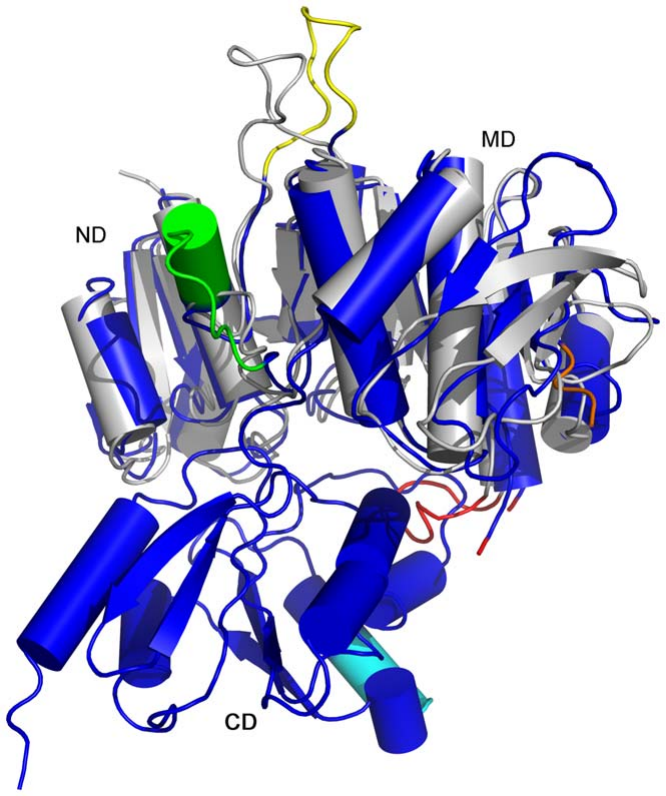
Comparison of crystal structures of full-length *Pa*Mpl and truncated *Nm*Mpl. The crystal structure of truncated *Nm*Mpl (grey, ND and MD only, PDB id 3eag) is similar to *Pa*Mpl (blue) and can be superimposed with an r.m.s.d. of 2.2 Å over 311 Cα atoms with a sequence identity of 57%. The main differences are: *Nm*Mpl lacks the *Pa*Mpl-specific sequence segments 141–152 (green), 262–285 (orange) and 444–450 (cyan); *Nm*Mpl residues 152–163 are positioned differently compared to the corresponding residues 161–171 in *Pa*Mpl (yellow); and the segment corresponding to a loop (residues 199–208, red) in *Nm*Mpl is disordered in *Pa*Mpl (residues 210–215, flanking residues 209 and 216 are in red).

A superimposition of *Pa*Mpl onto the partial structure of the *Nm*Mpl (ND and MD, PDB id 3eag) shows that the corresponding domains are quite similar with an r.m.s.d. of 2.2 Å over 311 Cα residues and a structure-based sequence identity of 57% (Figure 4). However, a few notable differences, for example, include *Pa*Mpl residues 141–152 (loop and helix, green), 262–285 (orange), and 444–450 (helix, cyan) that represent insertions into the core structure that are not present in *Nm*Mpl, the 161–171 loop (yellow), which adopts a different conformation compared to the corresponding loop (153–162) in *Nm*Mpl, and a disordered region in *Pa*Mpl (210–215) that is ordered (loop 199–208, red) in *Nm*Mpl.

**Figure 5:**
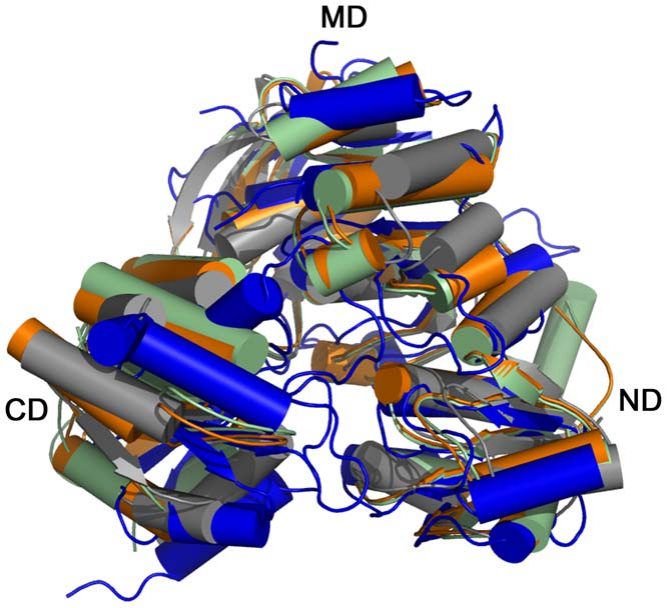
Comparison of *Pa*Mpl and MurC structures. Superimposition of *Pa*Mpl (blue) with *Ec*MurC (PDB 2f00, pale green), *Hi*MurC (PDB 1p31, orange), and *Tm*MurC (PDB 1j6u, grey) reveals a common structural core of 355 Cα residues with an r.m.s.d. of 2.7 Å.

**Figure 6:**
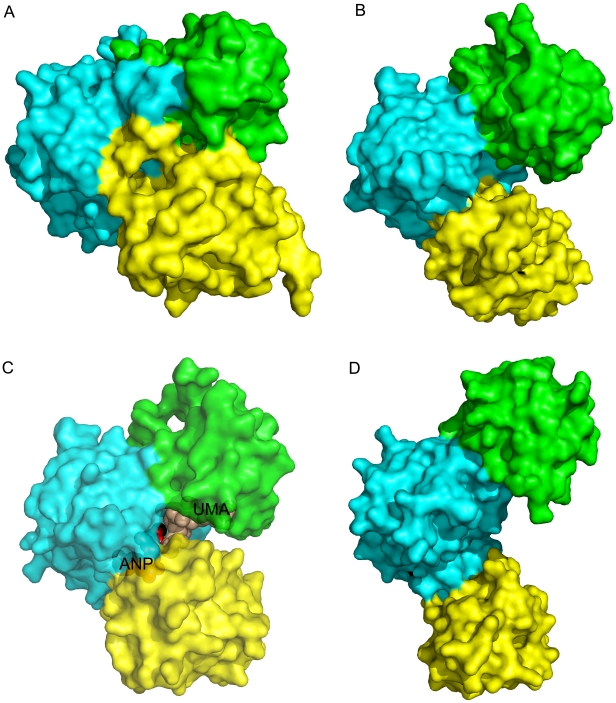
Surface representation of different domain dispositions in Mur family structures. Crystal structures of: (A) apo-*Pa*Mpl, (B) *Ec*MurC with bound Mg^2+^, (C) *Hi*MurC bound to substrates UDP-MurNAc-L-Ala (UMA) and AMPPNP (ANP), and metal; and (D) apo-*Ec*MurF (on slightly smaller scale compared to A–C as more extended conformation). For all proteins, ND is in green, MD is in cyan and CD is in yellow. These examples illustrate the conformational variability of these Mur enzymes. All molecules are in the same orientation based on their superimposition on the *Pa*Mpl MD. The CD of the apo-*Pa*Mpl is rotated 30° with respect to ND and MD compared to the MurC structure. The *Pa*Mpl domains may open up during substrate binding and close during catalysis.

As Mpl is most similar in sequence and function to MurC and since MurC-F enzymes have been compared in detail elsewhere [7], [8], [58], we have restricted our comparisons to MurC structures (*Ec*MurC with bound Mg^2+^, PDB id 2f00 and *Hi*MurC with substrate analogs UDP-MurNAc-L-Ala (UMA) and a nonhydrolyzable ATP analog AMPPNP (ANP) bound, PDB id 1p3d). *Pa*Mpl and *Ec*MurC can be superposed with an r.m.s.d. of 2.2 Å over 283 Cα atoms (representing the most structurally conserved regions, ND and MD, as there is variation in the orientation of CD). Residues in *Ec*MurC that correspond to the 3 domains of *Pa*Mpl are 1–118, 119–325 and 326–483, respectively. *Pa*Mpl is larger than *Ec*MurC, with major insertions in the MD (255 vs. 207 residues in the MD). In a domain pair-wise comparison, the respective ND, MD and CD superpose with r.m.s.d.s of 1.4 Å (26% structure-based sequence identity), 2.1 Å (33% sequence identity) and 2.1 Å (20% sequence identity) over 99, 195 and 125 Cα residues respectively. Flexible, multiple structure alignment of *Pa*Mpl with *Ec*MurC (PDB 2f00), *Hi*MurC (PDB 1p31), and *Tm*MurC (PDB 1j6u) using POSA [59] shows that these proteins share a common structural core (Figure 5) consisting of 355 Cα residues with an r.m.s.d. of 2.7 Å. Moreover, conformational flexibility is inherent in the overall architecture of Mur enzymes as illustrated by comparing the substrate-bound and apo structures of MurC and MurF (Figure 6, C and D, respectively). Superposition of the apo *Pa*Mpl structure with the substrate-bound structures of *Hi*MurC (PDB 1p3d and 1gqy) results in r.m.s.d.'s of 4.2 Å over 411 Cα residues and 4.9 Å over 414 Cα residues, as a result of significant domain movements. The largest conformational difference is in the disposition of the CD in Mpl, which is rotated 30° relative to ND and MD, when compared to its disposition in MurC (Figure 6, A and C, respectively). Detailed inspection of the individual domains reveals that the region with the least sequence conservation between Mpl and MurC proteins is in the CD as discussed later.

### Oligomerization

**Figure 7:**
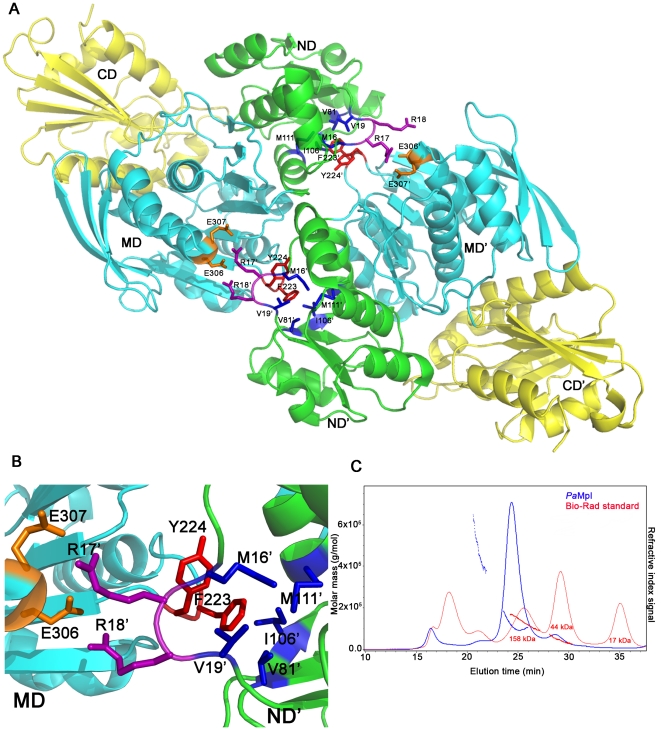
Oligomerization states of *Ec*MurC and *Pa*Mpl. (A) The dimer observed in the crystal structure of *Ec*MurC (PDB 2f00) with the domains from each monomer labelled ND, MD, CD, and ND', MD', CD'; respectively, and the residues involved in dimer interactions highlighted as sticks. (B) A detailed view of the residues involved in intermolecular contacts: F223 and Y224 from the MD of one protomer interact with M16', V19', V81', I106' and M111' from ND' of the other protomer. E306 and E307 from one protomer interact with R17' and R18' from the other protomer. (C) Profile of the SEC/SLS experiment displaying the refractive index signal (SEC, continuous trace) against elution time (minutes) for *Pa*Mpl (blue) compared with a molecular weight standard (red, BioRad gel filtration standard with 158, 44 and 17 kDa peaks representing bovine γ-globulin, chicken ovalbumin and horse myoglobin, respectively). The discontinuous trace represents the molar mass (g/mol) calculated by SLS. For *Pa*Mpl, the SEC profile suggested a tetrameric form in comparison to the standard, but the more accurate molar mass estimated from SLS averaged across the majority of the peak was 120.5 kDa (as calculated by the ASTRA software (Wyatt Technology), with an oligomer number of 2.09, indicating a dimer as the dominant species in solution.

*Pa*Mpl is present as a monomer in the crystal asymmetric unit and crystal packing analysis using PISA [41] did not identify any potential higher order oligomers in the crystal lattice. Analytical SEC suggested a tetramer in solution (Figure 7C, highest peak in the continuous blue curve). However, static light scattering measurements indicated a molecular weight of 120.5 kDa (Figure 7C, discontinuous blue curve) with an oligomeric state of 2.09, indicating that a dimer is the prominent oligomeric form in solution (n.b. in cases of discrepancy between SEC and light scattering, the light scattering results are more likely to represent the dominant solution state). SEC also indicated that *Ec*Mpl is dimeric in solution. In contrast, *Ec*MurD (PDB 1uag), *Ec*MurE (PDB 1e8c) and *Ec*MurF (PDB 1gg4), have been reported to be monomers in the crystal and in solution. Interestingly, *Ec*MurC (PDB 2f00) is also a dimer in the crystal structure but, in solution, displays a dynamic equilibrium between monomeric and dimeric forms. Both forms were shown to be active [16], [60]. The PFYG (residues 222–225) motif in *Ec*MurC (corresponding to PSTG, residues 232–235, in *Pa*Mpl), which is conserved in ∼50% of MurC proteins, is involved in dimerization. Phe223 and Tyr224 (in the H7-β11 loop) from MD of one *Ec*MurC monomer interact with Met16 and Val19 (not found in Mpl) and Val81, Ile106 and Met111 (corresponding positions in Mpl are Leu64, Thr89 and Phe94) in a hydrophobic pocket in the ND of its dimer partner (Figure 7, A and B). Also, Arg17 and Arg18 of *Ec*MurC, which form salt bridges with Glu306 and Glu307 respectively (Figure 7, A and B), are not found in *Pa*Mpl. Thus, residues involved in dimerization in *Pa*Mpl (and Mpl proteins in general) differ, suggesting that the mode of dimerization may also be different.

### ND:UDP-MurNAc binding region and MD:ATP and magnesium binding region

**Figure 8:**
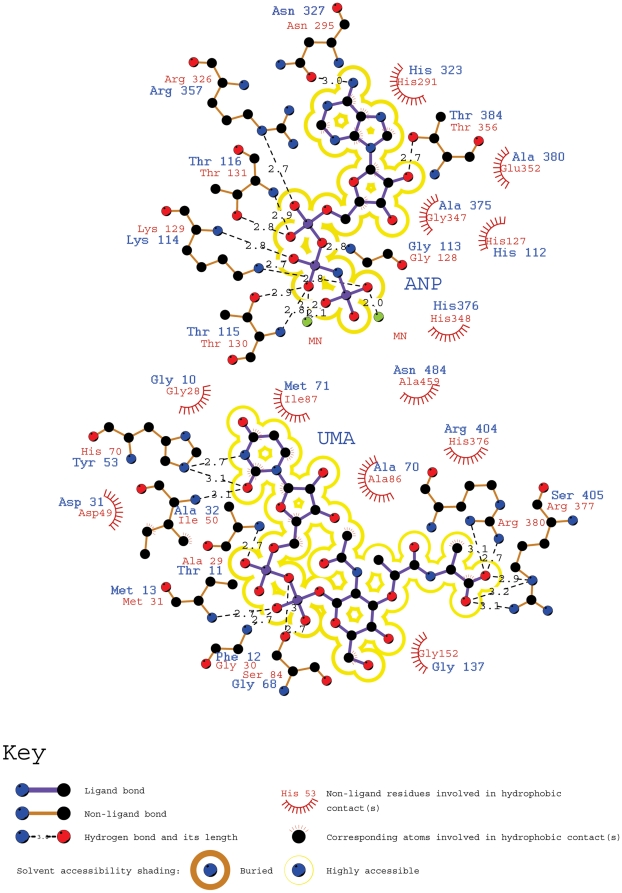
*Pa*Mpl residues potentially involved in interactions with ATP and UDP-MurNAc. Schematic representation rendered using LigPlot [61] of the interactions observed between *Hi*MurC and ligands UDP-MurNAc-L-Ala (UMA) and AMPPNP (ANP) in the crystal structure (PDB id 1p3d). The majority of these residues in *Hi*MurC (red labels) ND and MD are structurally conserved in *Pa*Mpl (blue labels), suggesting that Mpl and MurC have similar interactions with ATP and UDP-MurNAc.

Residues in the ND and MD that are important for interaction with UDP-MurNAc, ATP and metal have been described for Mur enzymes, and most of these residues are conserved in Mpl (Figure 2). Analysis of protein-ligand interactions observed in the *Hi*MurC/UMA/ANP complex structure using LigPlot [61] shows that many of the important residues are also structurally conserved in *Pa*Mpl (Figure 8).

**Figure 9:**
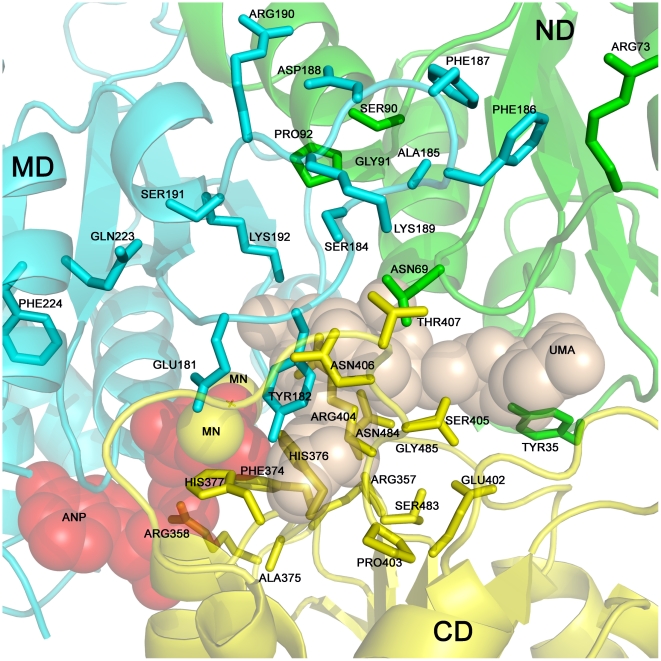
Additional Mpl-specific residues in ND, MD and CD that may be involved in substrate interactions. Model of *Pa*Mpl bound to ANP, UMA and metal based on the superimposition of the ND and MD of *Pa*Mpl onto the corresponding domains from *Hi*MurC (ND and MD are the most conserved domains between the proteins) bound to ANP and UMA (PDB id 1p3d). After superimposition, the ligand coordinates were transferred from *Hi*MurC to generate the model of *Pa*Mpl bound to ligands. Numerous residues in the binding pocket are unique and conserved in Mpl proteins and could be involved in additional interactions with substrates. These include ND Tyr35, Asn69, Arg73 and Gly74 (not shown). Ser90, Gly91 and Pro92 pack against Mpl-specific loop consisting of Ser184 (Thr or Cys in different Mpl proteins), Ala185, Phe186, Phe187, Asp188 and Lys189. Tyr182, which is directly upstream of this loop, could also be involved in the recognition. Mpl-specific residues in CD are likely to play a crucial role in substrate specificity and include Arg357, Arg358, Phe374, Ala375, His376, His377, Glu402, Pro403, Arg404, Ser405 (in dual conformation), Asn406, Thr407, Ser483 (in dual conformation), Asn484 and Gly485.

The glycine-rich loop GI(C/G)GTFM conserved across all Mpl enzymes (Figure 2, blue bar, residues 7–13 in *Pa*Mpl) is also present in MurC and other oxidoreductases [62], where it interacts with UDP. However, the Phe at position 12 in *Pa*Mpl is considerably larger than the corresponding glycine in MurC enzymes. Although, the identity of the three residues that interact with the uracil ring of UDP (*Hi*MurC Ile50, Ile87 and His70; *Ec*MurC Leu51, Ile88 and His71 [16]) is not conserved in Mpl (Ala32, Met71 and Tyr53 in *Pa*Mpl), the nature of the interactions is conserved (main chain, hydrophobic and side-chain hydrogen bond interactions with these 3 residues, respectively, Figure 8), as the *Pa*Mpl does also bind to UDP as demonstrated by biochemical characterization. Asp31 in *Pa*Mpl is conserved in almost all Mur enzymes and is important for interactions with the diphosphate of UDP (Figure 2, blue circle). Conserved residues in Mpl proteins that are also in the vicinity of the UDP-MurNAc-binding pocket, but are not structurally equivalent to those observed to interact with ligands in the *Hi*MurC structure (Figure 8), are Tyr35, Asn69, Arg73 and Gly74, which could also be involved in recognition and binding of UDP-MurNAc in Mpl (Figure 9). In addition, the Mpl-specific region, which is conserved in the ND, comprises of SGP (residues 90–92 in *Pa*Mpl) that packs against another conserved loop (S/T)AFFDKRSK (residues 184–192, Figure 2, green and black bars). These interactions could play a role in the different positioning of ND and MD (so as to interact with both the UDP and MurNAc moieties) compared to that in Mur enzymes (Figure 9). Tyr182, which is unique to Mpl (Figure 2, blue circle and Figure 9) and located just prior to the start of this loop, could also be involved in substrate recognition. Thus, the various Mpl residues described above are potentially involved in UDP-MurNAc interactions and provide a guide for site-directed mutagenesis to test their functional roles.

As for all other Mur enzymes, it is assumed that the ligation of peptide to UDP-MurNAc should also be ATP-driven in Mpl and that phosphorylation of the C-terminal carboxylate forms an acyl phosphate intermediate, followed by the nucleophilic attack of amino group of the amino acid or peptide substrate. Thus, in *Pa*Mpl, the corresponding adenine binding residues would be Asn205, His323 and Asn327 (Figure 2, magenta circles), the ribose binding residue would be Asp373 (Figure 2, orange circle), and residues that stabilize the tri-phosphate would be the GKTT motif (P-loop found in kinases and ATPases, residues 113–116; Lys 114 corresponding to Lys129 in *Hi*MurC and Lys130 in *Ec*MurC) (Figure 2, cyan circles), and Arg358 and His376 (Figure 2, orange circles) [10]. These residues are all structurally conserved when compared to the *Hi*MurC ligand complex (Figure 8). Like MurC, *Pa*Mpl activity (see ***Optimal conditions for enzyme activity***) is dependent on magnesium (in other structures of Mur ligases, two Mg ions (Mg1, Mg2) per molecule are found), although no Mg^2+^ is observed in our structure. Mg1 would interact with the β- and γ-phosphates of ATP and Mg2 would interact with the γ-phosphate based on available MurC structures. In *Ec*MurC (PDB 2f00), only Mg2 was observed due to the absence of ATP, whereas both metals were observed in the complex structure of *Hi*MurC (PDB 1p3d). Thus, the binding site of Mg1 in *Pa*Mpl is likely to be comprised of Thr115 and Glu178 (corresponding to Thr130 and Glu173 in *Hi*MurC) with the remainder of the coordination sphere completed by two water molecules and two oxygen atoms from the phosphate groups of the ATP. The second metal binding site in *Ec*MurC and *Hi*MurC is around His199 and His198, respectively, with waters making up the remainder of the coordination sphere. The corresponding residue in *Pa*Mpl is His210, but this region (residues 210–215) is disordered in our structure, probably due to the absence of metal ions. A loop (residues 404–414) from CD in *Pa*Mpl is folded into part of the ATP and UDP-MurNAc binding site (Figure 9). A significant conformational change is likely to occur in this region upon binding substrates and cofactors and involves ordering and closing of the His210 region towards Mg2 and opening of the CD. In the truncated *Nm*Mpl, which lacks CD, the region corresponding to missing residues 210–215 in *Pa*Mpl is ordered even in the absence of bound metal. Conserved residues in Mpl proteins that are in the vicinity of the ATP-binding pocket, but are not equivalent to those observed to interact with ANP in *Hi*MurC (Figure 8), are Tyr182, Gln223 and Phe224 (which is a phenylalanine or tyrosine in MurC) (Figure 9). Phe224 corresponds to a lysine in MurD (Lys198 in *Ec*MurD), MurE and MurF that is post-translationally carbamoylated. Several experiments have demonstrated that this carbamoylated lysine is essential [63] as it aids in the stability of Mg2 binding. In MurC, absence of this lysine is compensated by presence of a glutamate that is also conserved in Mpl enzymes (*Pa*Mpl Glu181) (Figure 9).

### CD: tripeptide binding region

The CD of Mpl is functionally unique from MurC-F proteins since it primarily attaches a tripeptide to UDP-MurNAc (n.b. it can also attach a tetra or pentapeptide, but less efficiently). MurC residues believed to be involved in binding to the incoming amino acid are His376, Arg377, Arg380, Tyr346 and His348 as observed in the *Hi*MurC-product complex (PDB 1p3d) (Figure 8, n.b. Tyr346 is not seen in this representation). In the case of MurE, it is believed that Arg416 should be the main determinant for peptide selectivity [64]. The various Mur enzymes, as well as Mpl, have most variation in this CD domain (Figure 2), which reflects that they are all likely to have different interactions with the incoming peptide substrate, but this aspect of Mur function remains the least characterized. Conserved, solvent-exposed, Mpl-specific residues in the CD are likely to be instrumental in defining substrate specificity and include Arg357, Arg358, Phe374, Ala375, His376, His377, Glu402, Pro403, Arg404, Ser405, Asn406, Thr407, Ser483, Asn484 and Gly485, which may be important in binding tri, tetra, and pentapeptide substrates (Figure 9). Based on their location in the *Pa*Mpl structure, it seems likely that, if these residues were to interact with peptide, the CD should undergo a conformational change with respect to the ND and MD.

### Kinetic parameters and substrate specificity of PaMpl

The expression and purification of *Pa*Mpl for biochemical characterization was carried out as outlined in ***Materials and Methods***. The kinetic parameters of the *Pa*Mpl enzyme towards its three substrates were determined (Table 2). These values (*k_cat_* = 260±20 min^−1^; *K_m_* = 1.00±0.13 mM, 0.11±0.05 mM and 0.36±0.08 mM for UDP-MurNAc, ATP and L-Ala-γ-D-Glu-*meso*-A_2_pm, respectively) were generally similar to those previously determined with the *E. coli* ortholog (*k_cat_* = 290 min^−1^; *K_m_* = 0.2 mM and 0.1 mM for ATP and L-Ala-γ-D-Glu-*meso*-A_2_pm, respectively). The *K_m_* value obtained for UDP-MurNAc, however, was significantly higher (*K_m Ec_*_Mpl_ = 0.25 mM).

**Table 2:** Kinetic parameters of *Pa*Mpl a.

Substrate	Apparent *K_m_* (mM)	Apparent *k*_cat_ (min^−1^)	Apparent *k*_cat_/*K*_m_ (min^−1^/mM)
ATP	0.11±0.05	260±20	2400±1100
UDP-MurNAc	1.00±0.13	260±20	260±39
L-Ala-γ-D-Glu-*meso*-A_2_pm	0.36±0.08	260±20	720±170

a The concentrations of the fixed substrates were 5 mM for ATP, 0.4 mM for UDP-MurNAc and 0.25 mM for L-Ala-γ-D-Glu-*meso*-A_2_pm. The concentration ranges for the varying substrates were 0.05 to 3 mM for ATP, 0.1 to 0.6 mM for L-Ala-γ-D-Glu-*meso*-A_2_pm, and 0.1 to 2 mM for UDP-MurNAc.

The substrate specificity of *Pa*Mpl was also investigated. As shown in Table 3, tetrapeptide L-Ala-γ-D-Glu-*meso-*A_2_pm-D-Ala and pentapeptide L-Ala-γ-D-Glu-*meso-*A_2_pm-D-Ala-D-Ala were also accepted as substrates, but less efficiently. As the Michaelis-Menten plots were linear up to 0.25 mM, it was only possible to determine enzyme velocities at this concentration (*k* values for these substrates were 5.9±0.6 min^−1^ and 5.3±0.6 min^−1^, respectively). *Pa*Mpl activity was comparatively much lower with smaller substrates L-Ala and L-Ala-D-Glu (*k* = 0.7±0.1 min^−1^ and 1.4±0.2 min^−1^, respectively) and substitution of *meso-*A_2_pm by L-Lys in the tripeptide dramatically decreased enzyme activity (*k* = 0.25±0.05 min^−1^ and 260±20 min^−1^ for L-Lys- and *meso*-A_2_pm-containing tripeptides, respectively). A clear preference for A_2_pm at the third position of the peptide had already been observed with *Ec*Mpl, although to a lesser extent (*k*_cat_ values of the *E. coli* enzyme for these two tripeptides were 16 min^−1^ and 290 min^−1^, respectively) [13].

**Table 3:** Substrate specificity of *Pa*Mpl.

Substrate	*k* (min^−1^)
L-Ala-γ-D-Glu-*meso*-A_2_pm	260±20^a^
L-Ala-γ-D-Glu-*meso*-A_2_pm-D-Ala	5.9±0.6
L-Ala-γ-D-Glu-*meso*-A_2_pm-D-Ala-D-Ala	5.3±0.6
L-Ala	0.7±0.1
L-Ala-D-Glu	1.4±0.2
L-Ala-γ-D-Glu-L-Lys	0.25±0.05
L-Ala-γ-D-Glu-L-Lys-D-Ala	0.10±0.03
L-Ala-γ-D-Glu-L-Lys-D-Ala-D-Ala	ND^b^

The *k* (*v*/E) values were determined with 0.25 mM of *meso*-A_2_pm-containing peptides, 4 mM of L-lysine-containing peptides, and 15 mM of L-Ala or L-Ala-D-Glu.

a Apparent *k*_cat_ value.

b ND, no detectable product formation under the experimental conditions used.

### Optimal conditions for enzyme activity

**Figure 10:**
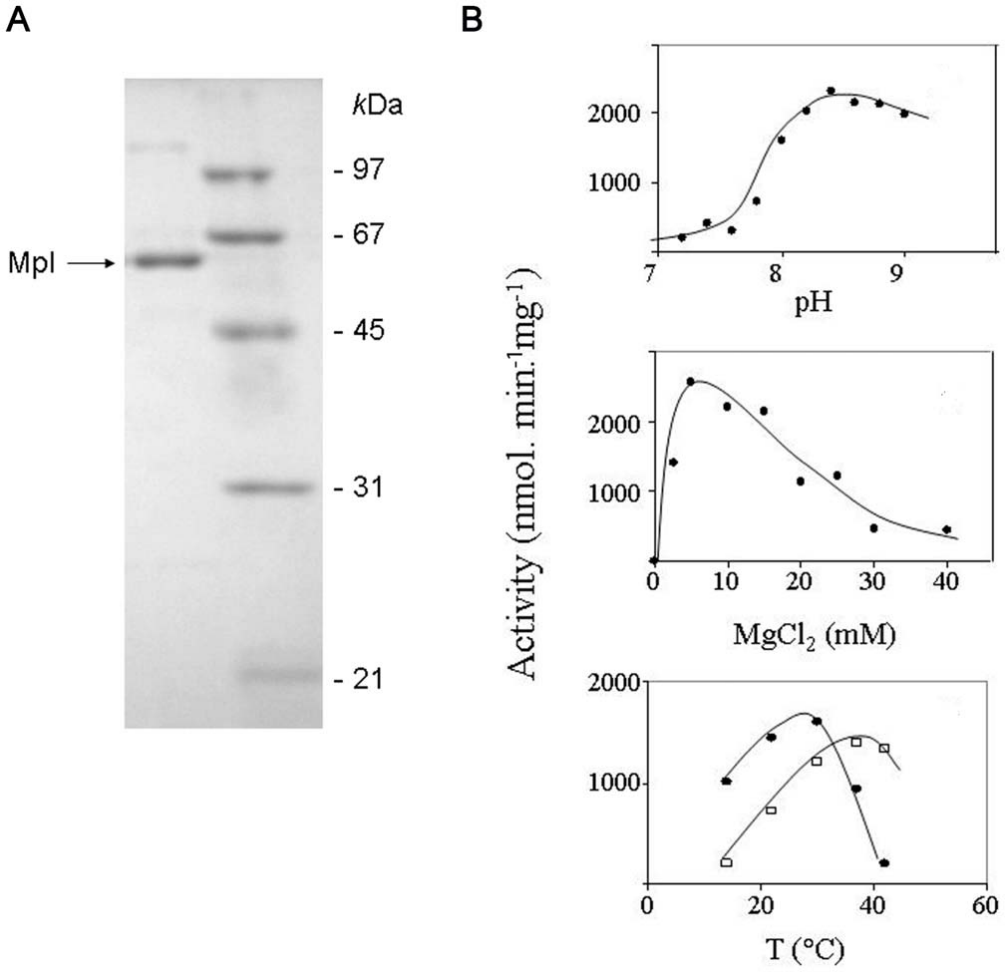
Expression and biochemical characterization of the *Pa*Mpl protein. (A) Protein expression and purification. Expression of the *Pampl* gene was induced in *E. coli* MLD2502 cells carrying the pSpeed ET::*Pampl* plasmid. The overproduced protein (His_6_-tagged form) was purified by two successive steps of chromatography on Ni^2+^-NTA agarose and Co^2+^ TALON resin. SDS-PAGE analysis of an aliquot of the final preparation is shown. Molecular mass standards (M) indicated on the right (kilodaltons) are phosphorylase b, 97; serum albumin, 66; ovalbumin, 45; carbonic anhydrase, 31; and trypsin inhibitor, 21. Staining was performed with Coomassie brilliant blue R250. (B) Effect of pH, Mg^2+^ and temperature on *Pa*Mpl activity. UDP-MurNAc:L-Ala-γ-D-Glu-*meso*-A_2_pm ligase activity of *Pa*Mpl (-•-) was determined at different pH values, Mg^2+^ concentrations, and temperatures. For the temperature profile, corresponding data obtained with the *Ec*Mpl ortholog are indicated for comparison (-□-). Assays were performed as described in ***Materials and Methods***, using 100 mM Tris-HCl buffer and concentrations of ATP, UDP-MurNAc and L-Ala-γ-D-Glu-*meso*-A_2_pm of 5.0 mM, 0.4 mM and 0.25 mM, respectively. The standard deviations were within ±5% of the values indicated.

Assessment of activity as a function of pH showed an optimal pH value of 8.4 (Figure 10). Like *Ec*Mpl and all other Mur ligases [13], [65], *Pa*Mpl requires a divalent cation for activity. The optimal concentration determined for Mg^2+^ was 5 mM (Figure 10). As *P. arcticus* is a psychrophilic Siberian permafrost bacterium, a comparative study of *Pa*Mpl and *Ec*Mpl activities as a function of temperature was performed (Figure 10). At a low temperature ∼15°C, the activity of *Pa*Mpl was almost twice that of *Ec*Mpl. The optimal temperatures for these two enzymes were 30°C and 37°C, respectively. The *Pa*Mpl activity was almost completely abolished at 42°C, although *Ec*Mpl still retained 95% of its optimal activity at this temperature.

## Discussion

The high-resolution crystal structure of the full-length *Pa*Mpl provides insights into the structure and relative orientations of the three domains in this cell wall recycling enzyme. Analysis of residues likely involved in binding UDP-MurNAc, ATP, metals and tri, tetra and pentapeptide substrates combined with enzyme kinetics and activity characterization, provide a basis for further experimentation to explore the structure and function of Mpl proteins. As a recycling enzyme, the role of Mpl is to attach the tripeptide L-Ala-γ-D-Glu-*meso*-A_2_pm to UDP-MurNAc. Structure analyses indicate that the orientation of the CD domain of *Pa*Mpl relative to the other domains is flexible and changes on substrate binding. The pre-catalytic form of Mpl must open up to allow UDP-MurNAc to bind to the ND, and the tripeptide has to approach and be recognized by the CD and positioned at the active site. *Pa*Mpl-specific residues in helix H16 may affect how the CD interacts with peptide substrate. Loop 161–171 that is near the domain boundary of the ND and MD is not found in *Ec*Mpl. As the protein domains separate to bind UDP-MurNAc, this *Pa*Mpl-specific loop may control the extent to which the ND can open and, in turn, may affect UDP-MurNAc binding. This conformational rearrangement may explain the higher *K*_m_ values of *Pa*Mpl for UDP-MurNAc and tripeptide. The *Pa*Mpl-specific segments 141–152, 161–171 and 262–285, which are not present in *Ec*Mpl, might be responsible for its relative sensitivity to higher temperature compared to *Ec*Mpl.

The Mur enzymes are established drug discovery targets as disruption of peptidoglycan biosynthesis is a validated path to bacterial cell death. The genes coding for the Mur ligases are essential for bacterial survival, as demonstrated by knockout experiments [3]. The recycling enzyme Mpl, although not essential for growth, could also be considered an interesting target for drug discovery. Indeed, deletion of the *mpl* gene and other genes involved in peptidoglycan recycling increases antibiotic susceptibility of some pathogenic strains. For instance, disruption of these non-essential genes in *Acinetobacter baylyi* results in at least a 10-fold reduction in the MIC of β-lactams [66]. The identification of the Mpl active site and the structural determinants that correlate with its function may lead to the design of better Mur inhibitors through structure-based drug design. Such compounds could then shutdown both the *de novo* and the recycling pathways for cell wall synthesis and act as novel antimicrobial agents. Alternatively, as recently discussed [13], the broad substrate specificity displayed by Mpl enzymes could potentially be exploited as an Achilles' heel for incorporation of toxic peptides into the peptidoglycan network.
